# Mental health and care utilization in survivors of adolescent and young adult cancer

**DOI:** 10.1093/jncics/pkad098

**Published:** 2023-11-20

**Authors:** Nikita V Baclig, Warren Scott Comulada, Patricia A Ganz

**Affiliations:** Department of Health Policy and Management, UCLA Fielding School of Public Health, Los Angeles, CA, USA; Division of Hematology and Oncology, Department of Medicine, David Geffen School of Medicine, University of California Los Angeles, Los Angeles, CA, USA; Department of Health Policy and Management, UCLA Fielding School of Public Health, Los Angeles, CA, USA; Department of Psychiatry and Biobehavioral Sciences, David Geffen School of Medicine, University of California Los Angeles, Los Angeles, CA, USA; Department of Health Policy and Management, UCLA Fielding School of Public Health, Los Angeles, CA, USA; Division of Hematology and Oncology, Department of Medicine, David Geffen School of Medicine, University of California Los Angeles, Los Angeles, CA, USA

## Abstract

**Background:**

Adolescent and young adult cancer survivors experience mental health challenges, yet little is known about the evolution of these difficulties. This study explored mental health symptoms and utilization among long-term adolescent and young adult cancer survivors.

**Methods:**

Using 30 432 respondents from the 2019 National Health Interview Survey, this study compared adults with a history of adolescent and young adult cancer (diagnosed when patients were between 15 and 39 years of age) to adults without adolescent and young adult cancer. Mental health symptom severity was measured using the Patient Health Questionnaire depression scale and 7-item Generalized Anxiety Disorder questionnaires. Care utilization constituted psychotherapy and mental health medication use. Inverse propensity score weights were used to balance demographics and combined with survey weights. Descriptive statistics, multivariable generalized linear models, and structural equation modeling with 2-sided tests were used for analysis.

**Results:**

We compared 639 adolescent and young adult survivors with 29 793 controls. Survivors were, on average, 20.5 years (SE = 0.74) past their cancer diagnosis dates. After adjusting for survey and propensity score weights, adolescent and young adult survivors reported more severe depression (incidence rate ratio = 1.42, 95% confidence interval [CI] = 1.09 to 1.84, *P* < .01) and anxiety (incidence rate ratio = 1.85, 95% CI = 1.55 to 2.21, *P* < .001). They were more likely to use psychotherapy (odds ratio = 1.91, 95% CI = 1.16 to 3.17, *P* < .05) and mental health medications (odds ratio = 1.89, 95% CI = 1.15 to 3.11, *P* < .05). Time since diagnosis was negatively associated with symptoms and utilization. Structural equation modeling demonstrated mediation of utilization effect by symptom severity.

**Conclusions:**

Adolescent and young adult survivors experience worse mental health in late survivorship, despite small improvements over time. We highlight the importance of survivorship care that addresses the long-term mental health needs of these survivors.

Adolescent and young adult patients with cancer are those diagnosed between the ages of 15 and 39 years. They have a unique cancer experience, with distinct patterns of incidence, more severe biology, and differential treatment outcomes compared with pediatric and older adult patients (1-4). They experience challenges in receipt of health care, are poorly represented in clinical trials, and deal with excess financial toxicity (5,6).

Adolescent and young adult patients experience important mental health sequelae of diagnosis and treatment (7). They self-identify as having poor mental health and have worse scores on distress scales (8,9). Poor perceived mental health has been linked to mental health diagnoses, and both are worse for adolescent and young adult patients than cancer-free or older oncology patients (10). Many reasons have been cited, including posttraumatic stress, fear of cancer recurrence, loss of productivity, tobacco and alcohol use, development of late toxicities, chronic comorbidities, and poor social support (11-17). Most studies have been conducted early in the survivorship experience, despite a relative survival rate of 85.5% in this population (18).

The adolescent and young adult mental health literature uses disparate assessment tools, restricting comparison, and frequently incorporates research rather than clinical metrics. This trend was highlighted in 2 recent reviews. Although Osmani and colleagues (19) estimated that 24% to 32% of adolescent and young adult survivors experience mental health consequences, Tanner et al. (20) reported mixed findings with regard to patient-reported mental health. Besides demonstrating that there remains equipoise concerning the effects of adolescent and young adult cancer on mental health, both reviews highlighted how few studies used clinically relevant scales.

The 7-item Generalized Anxiety Disorder (GAD-7) and Patient Health Questionnaire 9 (PHQ-9) are validated and clinically useful scales for assessing generalized anxiety and depressive disorders (21,22). Both instruments have been validated in cancer cohorts (23,24). An abbreviated version of the PHQ-9—the PHQ-8—is considered equivalent to the PHQ-9 (25,26). To date, the few studies that have used these tools in adolescent and young adult cancer survivors have applied them to small, specific cohorts (27-29).

The National Health Interview Survey (NHIS) is a cross-sectional survey of a representative sample of US households (30). In 2019, the NHIS underwent a redesign, incorporating the PHQ-8 and GAD-7 on a rotating schedule, thus providing nationally representative mental health data from robustly validated questionnaires. By also addressing a history of cancer diagnosis and current use of mental health care, the NHIS 2019 offered a unique opportunity to examine long-term mental health outcomes for adolescent and young adult survivors.

We aimed to examine how being a long-term adolescent and young adult cancer survivor affects mental health symptom severity and care utilization and to evaluate whether time since diagnosis affects this relationship. We hypothesized that symptom severity and utilization would be higher for adolescent and young adult survivors than for controls, that symptoms would mediate the effect of adolescent and young adult survivorship on utilization, and that time since diagnosis would decrease symptom severity and utilization for adolescent and young adult survivors.

## Methods

### Study design

This observational, cross-sectional study compared adult survivors of adolescent and young adult cancers with those individuals without a history of adolescent and young adult cancer, using survey data from the 2019 NHIS published by Integrated Public Use Microdata Series Health Surveys (31). The survey included 31 997 adults, selected using geographically clustered sampling techniques based on the decennial census (32).

### Patient eligibility

Survivors of adolescent and young adult cancer were identified as adults (aged ≥18 years) who responded “yes” to the survey question “Have you ever been told you had cancer?” and reported an age at diagnosis of 15 to 39 years when asked, “How old were you when you were first told you had [type] cancer?” This group was compared with all respondents who did not meet these criteria.

### Variable specification

We used the embedded PHQ-8 and GAD-7 questions to determine mental health symptom severity. Individual question responses ranged from “not at all” (0) to “nearly every day” (3), allowing for total scores to be calculated for depression (0-24) and anxiety (0-21) symptoms. Respondents with missing data for all GAD-7 questions or all PHQ-8 questions were excluded. Partially missing GAD-7 or PHQ-8 questions were counted as 0, though this was limited, affecting 100 observations (<0.5%) for both the GAD-7 and PHQ-8 instruments.

Surveyed adults were asked, “During the past 12 months, did you receive counseling or therapy from a mental health professional such as a psychiatrist, psychologist, psychiatric nurse or clinical social worker?” A “yes” response was considered as having used psychotherapy, “no” as not, and those who refused or chose “don’t know” (<2%) were considered missing. All adults were asked, “Do you take prescription medication for depression?” and “Do you take prescription medication for these feelings [referencing worried/nervous/anxious feelings]?” If the response to either question was “no,” “refused,” or “don’t know,” the adult was then asked, “During the past 12 months, did you take prescription medication to help with any other emotions or with your concentration, behavior or mental health?” Adults who responded “yes” to the original or follow-up question were considered as having used medications for mental health. If the adult responded “no” to all 3 questions, they were not. Other combinations were considered missing data (<2%).

Thorough literature review implicated several survivor characteristics as potential confounders, including age, sex, level of education, partner support, children, smoking status, number of comorbidities, income, disability status, health insurance coverage, and usual source of care (henceforth, “covariates”). Most covariates were taken directly from NHIS survey questions, though the number of comorbidities was derived from separate indicator variables for hypertension, high cholesterol, coronary heart disease, angina, heart attack, stroke, asthma, diabetes, chronic obstructive pulmonary disease, and arthritis. For each variable, unevaluable responses were recategorized to missing values. We calculated time since diagnosis by subtracting age at diagnosis from age at survey. For those without a cancer diagnosis (n = 28 108) and those missing age (n = 3) or cancer diagnosis data (n = 64), time since diagnosis was considered to be 0 years.

### Statistical weights and missing data

We used survey and propensity score weights to account for the survey data-collection mechanism and to balance sociodemographic characteristics between adolescent and young adult survivors and the general population. Data were survey weighted according to the sampling strategy specific to the 2019 NHIS survey (33). Inverse propensity score weights were calculated using demographic variables that differed between the exposure and comparison groups (age, sex, and race/ethnicity). We applied the product of survey and propensity score weights to regression analyses, as previously described (34). Variables used for propensity score calculation were excluded from statistical models. Missing data were minimal (<5% of observations), so observations with missing data were excluded.

### Statistical analysis

Statistical analysis was performed using Stata, version 15.1 (StataCorp LP, College Station, TX) and SAS, version 3.81 (SAS Institute Inc, Cary, NC) statistical software. Double-sided ɑ = .05 was used. Descriptive statistics and *t* or χ^2^ tests were used to compare outcome variables between adolescent and young adult survivors and the general population. Independent multivariable generalized linear models were used to calculate the effect of adolescent and young adult survivorship on outcomes. We selected negative binomial models to address the right-skew of the data distributions for the GAD-7 and PHQ-8 totals (Supplementary Figures 1 and 2, available online) and logistic regression models for the binary mental health-care utilization variables. Among adolescent and young adult cancer survivors, we estimated the effect of time since diagnosis on outcomes with the appropriate generalized linear models. Both unadjusted and adjusted models, which controlled for covariates not included in propensity score determination, were estimated for all dependent variables.

**Figure 1.**
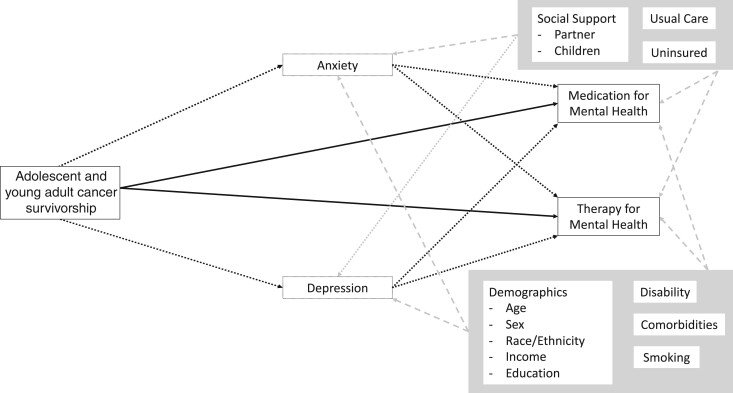
Conceptual model combining adolescent and young adult survivorship, mental health symptoms, and mental health-care utilization. Adolescent and young adult cancer survivorship is the binary independent variable. The dependent concept is health-care utilization, determined using 2 binary variables: medication for mental health (received mental health medication in the past 12 months) and therapy for mental health (received psychotherapy for mental health in the past 12 months). Anxiety and depression are mediators along the path from adolescent and young adult cancer survivorship to mental health-care utilization, as we hypothesized that these are major contributors to survivors’ intention to seek care. Both are used as count variables. Several additional concepts are implicated in adolescent and young adult survivors’ experience with mental health and were used here as confounding variables. Age; sex; race/ethnicity; social support, including having a partner or children; smoking status; comorbidities; disability; education level; insurance status; and usual source of care have demonstrated links to anxiety, depression, and care utilization. **Solid line box:** independent or dependent variable. **Dotted line box:** mediator variable. **Shaded box:** confounder. **Dotted line:** mediated (indirect) pathway. **Solid line:** residual (direct) pathway. **Dashed line:** confounding pathway.

Literature review prompted a conceptual model (Figure 1) that positioned depression and anxiety symptom severity as mediators of the effect of adolescent and young adult survivorship on mental health-care utilization. We tested this mediation using structural equation modeling. First, simultaneous total effects were estimated. Outcome variables were dichotomous and modelled using the Bernoulli family and logit link functions. Next, separate direct (residual) and indirect (mediated) effects were modelled by adding mediator variables (PHQ-8 and GAD-7). Mimicking prior analyses, we used the negative binomial family and the log link function for mediator variables. SEs and *P* values were calculated. Both unadjusted and adjusted models were run, controlling for all covariates not included in propensity score determination.

As survey questions addressing therapy and medication use queried the 12 months before the time of survey, we recognized the possibility of utilization preceding cancer diagnosis. An additional sensitivity analysis was therefore conducted excluding adolescent and young adult survivors with time since diagnosis less than 1 year (n = 15).

## Results

Demographic characteristics of the study cohort are shown in Table 1. In total, 30 432 adults were included, of whom 639 reported a history of adolescent and young adult cancer and 29 793 did not. Adolescent and young adult cancer survivors were older (49.8 vs 47.6 years), more likely to be female (71.2% vs 51.3%), and less ethnically diverse (77.9% vs 63.4% non-Hispanic White). Adolescent and young adult cancer survivors reported 3 or more comorbidities more frequently (23.1% vs 16.5%) and were more disabled (14.1% vs 8.7%). There were no large differences in educational attainment or income between the 2 groups, and both groups reported similar rates of partnership and children. Adolescent and young adult survivors were more likely to be current and former smokers (20.2% vs 13.8% and 25.6% vs 22.5%, respectively). Adolescent and young adult survivors did not demonstrate decreased access to care, with similar levels of insurance coverage (10.6% vs 11.5%) and lower rates of being without a usual source of care (6.7% vs 10.5%). After application of inverse propensity score weighting, the data demonstrated standardized differences for age, sex, and race/ethnicity between ‒0.25 and 0.25 (Supplementary Figure 3, available online).

**Table 1.** Basic characteristics, by survey-weighted frequencies and unweighted counts

	No adolescent and young adult cancer history		Adolescent and young adult cancer survivor	
	Weighted No. (%)	Unweighted sample, No.	Weighted No. (%)	Unweighted sample, No.
No.	—	29 793	—	639
Age, weighted mean (SE), y^a^	47.6 (0.17)	—	49.8 (0.79)	—
Female sex	51.3	15 924	71.2	478
Educational attainment				
Did not complete high school	12.2	2721	11.4	55
High school graduate or GED	27.2	7646	24	136
Some college	17.9	4885	16.6	108
Associate degree	13.3	3899	17.8	107
Bachelor’s degree	18.5	6535	19.1	142
Master’s degree	8.1	3059	9	72
Doctorate	2.8	1048	2.2	19
Race/ethnicity				
Hispanic	16.5	3871	12.7	58
Non-Hispanic Black	11.6	3198	5.2	33
Non-Hispanic White	63.4	20 443	77.9	520
Other^b^ and multiple	8.5	2281	4.2	28
Married or living with partner	59	15 261	59.6	303
Children in the home	33.3	8031	35.7	187
No. of comorbid conditions				
No comorbidities	44.6	11 774	33.5	202
1 comorbidity	24.7	7308	25.5	153
2 comorbidities	14.2	4721	18	117
≥3 comorbidities	16.5	5990	23.1	167
Smoking status				
Never smoker	63.7	18 138	54.2	328
Former smoker	22.5	7615	25.6	177
Current smoker	13.8	4040	20.2	134
Family income as a percentage above the federal poverty level				
0%-99%	10.9	3206	12.6	80
100%-199%	18.4	5319	20.3	127
200%-299%	16.8	4849	16	106
300%-399%	14.2	4123	10.8	70
400%-499%	10.4	3189	13.1	73
≥500%	29.2	9107	27.2	183
Positive disability using Washington Group Short Set Composite	8.7	3026	14.1	108
No health insurance coverage	11.5	2662	10.6	59
No usual source of care	10.5	2711	6.7	46
Years since cancer diagnosis,^a^ weighted mean (SE)	—	—	20.5 (0.74)	—
≤5 y since cancer diagnosis	—	—	19.1	107

a Values presented are not weighted frequencies but weighted mean (SE) because these reflect continuous (not categorical) variables.

b Includes non-Hispanic Asian, non-Hispanic American Indian or Alaska Native, and multiple races.

Adolescent and young adult survivors reported a weighted average of 20.5 years (SE = 0.74) since their cancer diagnoses, and more than 80% were more than 5 years past their diagnosis date (Supplementary Figure 4, available online). Average age at first cancer diagnosis was 29.31 years (SE = 0.35) (Supplementary Figure 5, available online). A wide variety of malignancy types was self-reported (Supplementary Table 1, available online). Gynecologic cancers (179 cases [26.5%]) were most frequent, of which cervical cancers were most common (145 cases [81.0%]). Distribution of malignancies otherwise represented population estimates (18), with slight overrepresentation of nonmelanoma (17.6%) and melanoma (11.7%) skin cancers.

Adolescent and young adult cancer survivors reported greater severity of anxiety and depression symptoms than those without a history of adolescent and young adult cancer. Compared with the general population, mean unadjusted GAD-7 score for adolescent and young adult survivors was 2.83 (95% confidence interval [CI] = 2.12 to 3.54) compared with 2.04 (95% CI = 1.97 to 2.10), and the mean PHQ-8 score was 3.37 (95% CI = 2.54 to 4.20) compared with 2.46 (95% CI = 2.39 to 2.53). Negative binomial incidence rate ratios showed that being an adolescent and young adult survivor was associated with 1.37 (95% CI = 1.09 to 1.72) and 1.39 (95% CI = 1.11 to 1.75) times higher PHQ-8 and GAD-7 scores than the general population (Table 2). When the models were adjusted for covariates, incidence rate ratios were further from the null (1.42 and 1.85 for PHQ-8 and GAD-7, respectively) and remained statistically significant (*P* < .01).

**Table 2.** Regression model output, with crude and adjusted incidence rate or odds ratios, according to the model used

Model	Independent variable	Dependent variable	No.^a^	Crude	Adjusted^b^
**Negative binomial models**				**Incidence rate ratio (95% confidence interval)**	
1	Adolescent and young adult survivorship	PHQ-8 total score	30 432	1.37 (1.09 to 1.72)^d^	1.42 (1.09 to 1.84)^d^
2	Adolescent and young adult survivorship	GAD-7 total score	30 432	1.39 (1.11 to 1.75)^d^	1.85 (1.55 to 2.21)^e^
3	Time since diagnosis	PHQ-8 total score	639	1.00 (0.99 to 1.00)	0.99 (0.98 to 0.99)^c^
4	Time since diagnosis	GAD-7 total score	639	0.98 (0.97 to 0.99)^d^	0.98 (0.97 to 0.99)^e^
**Logistic regression models**				**Odds ratio (95% confidence interval)**	
5	Adolescent and young adult survivorship	Therapy use	30 432	1.25 (0.90 to 1.75)	1.91 (1.16 to 3.17)^c^
6	Adolescent and young adult survivorship	Medication use	30 432	1.37 (1.02 to 1.84)^c^	1.89 (1.15 to 3.11)^c^
7	Time since diagnosis	Therapy use	639	0.99 (0.97 to 1.01)	0.96 (0.93 to 0.98)^d^
8	Time since diagnosis	Medication use	639	1.00 (0.98 to 1.02)	0.97 (0.94 to 0.99)^d^

a Models 1, 2, 5, and 6 were run using the entire sample of complete cases. Models 3, 4, 7, and 8 were run using just adolescent and young adult survivors (n = 639). GAD-7 = 7-item Generalized Anxiety Disorder; PHQ-8 = Patient Health Questionnaire depression scale.

b Models 1, 2, 5, and 6 were adjusted for income, education level, insurance status, usual source of care, number of comorbidities, disability status, smoking status, partner status, children, and time since diagnosis. Models 3, 4, 7, and 8 were adjusted for all the above variables, except time since diagnosis (primary regressor for these models).

c *P* < .05.

d *P* < .01.

e *P* < .001.

Adolescent and young adult cancer survivors also reported greater mental health-care utilization. In total, 20.4% of adolescent and young adult cancer survivors compared with 15.7% of the general population reported taking mental health–related medications in the past 12 months (*P* = .03). There was no statistically significant difference in the use of psychotherapy, however, with 11.6% of adolescent and young adult survivors and 9.5% of the general population reporting use (*P* = .18). From logistic regression (Table 2), we found that compared with the general population, being an adolescent and young adult survivor was associated with 1.37 (95% CI = 1.02 to 1.84) times greater odds of taking medication for mental health. Adjusting for covariates strengthened this association (odds ratio = 1.89) while maintaining statistical significance. Adolescent and young adult survivorship did not show a statistically significant association with therapy use in our unadjusted model (odds ratio = 1.25, 95% CI = 0.90 to 1.75), but after adjusting for covariates, it was associated with 1.91 times greater odds of using psychotherapy (95% CI = 1.16 to 3.17).

Among adolescent and young adult survivors, time since diagnosis had a small, negative, and statistically significant association with mental health symptom severity and care utilization. Using negative binomial models, we estimated that a 1-year increase in time since diagnosis was associated with a 2% decrease in GAD-7 score (crude incidence rate ratio = 0.98, 95% CI = 0.97 to 0.99). Unadjusted estimates for PHQ-8 score were not statistically significantly different from zero, but when adjusted for covariates, there was a statistically significant negative association between time since diagnosis for both GAD-7 score (incidence rate ratio = 0.98, 95% CI = 0.97 to 0.99) and PHQ-8 score (incidence rate ratio = 0.99, 95% CI = 0.98 to 0.99). Time since diagnosis was similarly associated with a small negative effect on the use of psychotherapy and medication. Controlling for covariates, a 1-year increase in time since diagnosis was associated with a 4% and a 3% decrease in the odds of using psychotherapy or medication, respectively (Table 2).

We then used structural equation modeling to conduct a mediation analysis. First, a total effects model was estimated that included the independent variable (adolescent and young adult survivorship) and 2 dependent variables (therapy and medication use). This simultaneous model, run both unadjusted and adjusted for covariates, produced similar estimates to our prior individual logistic regressions (Table 2, models 5 and 6).

**Figure 2.**
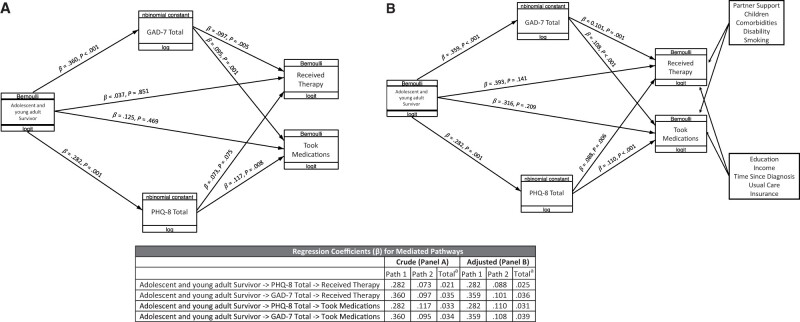
Structural equation models of adolescent and young adult cancer survivorship, symptom severity, and care utilization. **A**) Unadjusted structural equation model with mediated (indirect) and residual (direct) pathways from adolescent and young adult survivor (adolescent and young adult cancer survivors [referent is no history of adolescent and young adult cancer]) to health-care utilization variables; received therapy (received psychotherapy in the past 12 months [referent is no psychotherapy]); and took medications (received mental health medications in the past 12 months [referent is no medication use]). Individual variables are labelled at the top with generalized linear model family (eg, Bernoulli) and labelled at the bottom with the link function (eg, logit). β values represent generalized linear model regression coefficients with associated *P* values. **B**) Structural equation modeling was adjusted for select covariates. The table reflects the total regression coefficients for combined residual pathways. Total regression coefficient values were calculated according to path 1 × path 2 = total. GAD-7 = 7-item Generalized Anxiety Disorder; PHQ-8 = Patient Health Questionnaire depression scale.

Next, we estimated a structural equation modeling with GAD-7 and PHQ-8 as mediators of the effect of adolescent and young adult survivorship on mental health utilization (Figure 2). The direct (residual) effects of adolescent and young adult survivorship on the log odds of taking medication and using therapy were small and positive but statistically nonsignificant (β = .125, *P* = .469 and β = .037, *P* = .851, respectively). Indirect (mediated) pathways combine effects of the independent variable on a mediator variable with the effects of that mediator on the dependent variable. The total effect through this indirect pathway is expressed as the product of the 2 regression coefficients. All indirect pathways demonstrated statistically significant positive coefficients of similar magnitudes, except for the path connecting PHQ-8 score to therapy use (β = .073, *P* = .075). After adjusting for covariates, however, this path demonstrated statistical significance (Figure 2). The adjusted model was otherwise similar to the unadjusted model, with all indirect (mediated) pathways maintaining statistically significant regression coefficients and all direct (residual) pathways demonstrating statistically nonsignificant coefficients (β = .393, *P* = .141 and β = .316, *P* = .209 for therapy and medication use, respectively). Thus, the effects of adolescent and young adult survivorship on mental health-care utilization are shown to be mediated by severity of anxiety and depression.

As a sensitivity analysis, all models were then run, excluding survey respondents with cancer diagnosis less than 1 year in the past (n = 15), without meaninful changes in results (Supplementary Table 2, Supplementary Figure 6, available online).

## Discussion

We used a nationally representative sample to explore mental health symptom severity and mental health-care utilization among long-term adolescent and young adult survivors. Several decades into survivorship, these survivors reported more anxiety and depression than the general population and were more likely to use psychotherapy and mental health medications. These findings were robust to the passage of time, which had a statistically significant but small effect on reducing symptom severity and utilization. The effect of adolescent and young adult survivorship on mental health-care utilization was mediated through mental health symptom severity.

This study is the first to report ongoing mental health disturbances, on average, 20.5 years after an adolescent and young adult cancer diagnosis. These cross-sectional analyses took advantage of the temporal relationship between a reported history of adolescent and young adult cancer diagnosis and present-day symptom severity and utilization. Although a cross-sectional study cannot suggest causation, the analytic strategy we used, incorporating time since diagnosis, overcomes the logistical and financial challenges associated with conducting a long-term, prospective cohort study. Although the latter would provide stronger information about causality, such studies are threatened by participant selection bias, lack of a comparative control group, and attrition from longitudinal follow-up.

The findings reported here are strengthened by the PHQ-8 and GAD-7 assessments, clinically relevant tools that have not been previously applied to a US cohort of adolescent and young adult survivors. Confirming that mental health is worse for long-term adolescent and young adult survivors is novel and can be used as a benchmark for future research and clinical practice. Further, these analyses are among the first to use structural equation modeling to explore mediation between mental health symptoms and utilization. With statistically significant mediated and nonsignificant residual pathways, we demonstrated that mental health-care utilization is tied to symptoms of anxiety and depression. Although this finding may be expected, it reinforces the importance of screening for anxiety and depression to ensure that symptomatic individuals are receiving appropriate care.

The findings of this study have important implications for health policy. Although guidelines suggest that survivorship care continue for the remainder of a cancer patient’s life (35,36), for most individuals, survivorship is measured in years, not decades. For adolescent and young adult cancer survivors, many of whom live a normal lifespan, survivorship can last 50 to 60 years. Follow-up care should include regular symptom screening with validated tools such as the PHQ-8 and GAD-7, as recommended by the recent update on management of anxiety and depression in adult cancer survivors (37). Our study demonstrates average scores that fall below clinical cutoffs, but averages mask individuals who report much greater symptomatology. We would expect some proportion of long-term adolescent and young adult survivors to experience severe symptoms that require attention. Given the low cost of mental health screening and its guideline recommendations in the general population (38,39), efforts should be made to ensure that adolescent and young adult cancer survivors are screened and referred for mental health services., Our study results underscore the persistence of increased symptoms decades after cancer diagnosis.

Despite drawing data from a large national survey, our findings are limited by a small adolescent and young adult survivor cohort. This limitation was not unexpected, given that adolescent and young adult cancers make up 4.5% of new cancer cases annually (18). Adolescent and young adult cancer survivors made up only 2.1% of adults in NHIS 2019 because the survey sampling strategy was not designed around cancer incidence. Our study was also limited to a single year of data because the PHQ-8 and GAD-7 questions are not included in the NHIS survey every year. With data from future years of the NHIS, a larger sample may allow additional analyses.

The comparison group in these analyses included individuals without a history of cancer as well as those who may have had cancers diagnosed in older adulthood (≥40 years of age). Given small numbers (<10% of the comparison group) and the heterogeneity of older adult cancer survivors, additional analysis of this cohort was not pursued. An additional limitation is the potential for selection bias inherent in survey-based research. Although it is plausible that increased psychological symptom severity was associated with survey nonresponse, the inclusion of data from these individuals would have strengthened our findings. Finally, the secondary nature of our data sources limits our evaluation of mental health symptom severity, diagnoses, or care utilization before cancer diagnosis.

Despite these limitations, our findings of excess anxiety and depression in adolescent and young adult cancer survivors with standardized clinical screening tools decades after a cancer diagnosis call out the need for further investigation of this issue in this at-risk population. Our findings also validate the association between symptom severity and services. Future research should work to improve adherence to mental health screening and how best to intervene to ensure adequate care for all survivors of adolescent and young adult cancer.

## Supplementary Material

pkad098_Supplementary_Data

## Data Availability

The data underlying this article are publicly available at Integrated Public Use Microdata Series Health Surveys: NHIS*,* at https://nhis.ipums.org/nhis/. Data analysis scripts that generated the results are available on request.

## References

[1] Coccia PF. Overview of adolescent and young adult oncology. J Oncol Pract. 2019;15(5):235-237. doi:10.1200/JOP.19.00075 31009282

[2] Bleyer WA. Cancer in older adolescents and young adults: epidemiology, diagnosis, treatment, survival, and importance of clinical trials. Med Pediatr Oncol. 2002;38(1):1-10. doi:10.1002/mpo.1257 11835231

[3] Tricoli JV , Blair DG, Anders CK, et al Biologic and clinical characteristics of adolescent and young adult cancers: acute lymphoblastic leukemia, colorectal cancer, breast cancer, melanoma, and sarcoma. Cancer. 2016;122(7):1017-1028. doi:10.1002/cncr.29871 26849082 PMC4803597

[4] U.S. Department of Health and Human Services, National Institutes of Health, National Cancer Institute, LIVESTRONG Young Adult Alliance. Closing the Gap: Research and Care Imperatives for Adolescents and Young Adults with Cancer. Report of the Adolescent and Young Adult Oncology Progress Review Group. ayao_prg_report_2006_final.pdf. https://www.livestrong.org/sites/default/files/what-we-do/reports/ayao_prg_report_2006_final.pdf. Accessed October 23, 2021.

[5] Osborn M , Johnson R, Thompson K, et al Models of care for adolescent and young adult cancer programs. Pediatr Blood Cancer. 2019;66(12):e27991. doi:10.1002/pbc.27991 31524328

[6] Lu AD , Zheng Z, Han X, et al Medical financial hardship in survivors of adolescent and young adult cancer in the United States. JNCI J Natl Cancer Inst. 2021;113(8):997-1004. doi:10.1093/jnci/djab013 33839786 PMC8328985

[7] Rosgen BK , Moss SJ, Fiest KM, et al Psychiatric disorder incidence among adolescents and young adults aged 15-39 with cancer: population-based cohort. JNCI Cancer Spectr. 2022;6(6):pkac077. doi:10.1093/jncics/pkac077 36321955 PMC9733973

[8] Tai E , Buchanan N, Townsend J, Fairley T, Moore A, Richardson LC. Health status of adolescent and young adult cancer survivors. Cancer. 2012;118(19):4884-4891. doi:10.1002/cncr.27445 22688896 PMC5292773

[9] Kaul S , Avila JC, Mutambudzi M, Russell H, Kirchhoff AC, Schwartz CL. Mental distress and health care use among survivors of adolescent and young adult cancer: a cross-sectional analysis of the National Health Interview Survey. Cancer. 2017;123(5):869-878. doi:10.1002/cncr.30417 27859009

[10] Lang MJ , Giese-Davis J, Patton SB, Campbell DJT. Does age matter? Comparing post-treatment psychosocial outcomes in young adult and older adult cancer survivors with their cancer-free peers. Psychooncology. 2018;27(5):1404-1411. doi:10.1002/pon.4490 28672093

[11] McCarthy MC , McNeil R, Drew S, et al Psychological distress and posttraumatic stress symptoms in adolescents and young adults with cancer and their parents. J Adolesc Young Adult Oncol. 2016;5(4):322-329. doi:10.1089/jayao.2016.0015 27214245

[12] Kwak M , Zebrack BJ, Meeske KA, et al Prevalence and predictors of post-traumatic stress symptoms in adolescent and young adult cancer survivors: a 1-year follow-up study. Psychooncology. 2013;22(8):1798-1806. doi:10.1002/pon.3217 23135830

[13] Yang Y , Li W, Wen Y, et al Fear of cancer recurrence in adolescent and young adult cancer survivors: a systematic review of the literature. Psychooncology. 2019;28(4):675-686. doi:10.1002/pon.5013 30703261

[14] Zebrack BJ , Corbett V, Embry L, et al Psychological distress and unsatisfied need for psychosocial support in adolescent and young adult cancer patients during the first year following diagnosis. Psychooncology. 2014;23(11):1267-1275. doi:10.1002/pon.3533 24664958

[15] Asvat Y , King AC, Smith LJ, Lin X, Hedeker D, Henderson TO. Substance use behaviors in adolescent and young adult cancer patients: associations with mental and physical health. Psychooncology. 2020;29(6):1068-1076. doi:10.1002/pon.5378 32154963

[16] Ryder-Burbidge C , Diaz RL, Barr RD, et al The burden of late effects and related risk factors in adolescent and young adult cancer survivors: a scoping review. Cancers. 2021;13(19):4870. doi:10.3390/cancers13194870 34638350 PMC8508204

[17] Sansom-Daly UM , Wakefield CE. Distress and adjustment among adolescents and young adults with cancer: an empirical and conceptual review. Transl Pediatr. 2013;2(4):167-197. doi:10.3978/j.issn.2224-4336.2013.10.06 26835313 PMC4729076

[18] SEER. Cancer Among Adolescents and Young Adults (AYAs) - Cancer Stat Facts. https://seer.cancer.gov/statfacts/html/aya.html. Accessed November 30, 2021.

[19] Osmani V , Hörner L, Klug SJ, Tanaka LF. Prevalence and risk of psychological distress, anxiety and depression in adolescent and young adult (AYA) cancer survivors: a systematic review and meta-analysis. Cancer Med. 2023;12(17):18354-18367. doi:10.1002/cam4.6435 37559504 PMC10523984

[20] Tanner S , Engstrom T, Lee WR, et al Mental health patient-reported outcomes among adolescents and young adult cancer survivors: a systematic review. Cancer Med. 2023;12(17):18381-18393. doi:10.1002/cam4.6444 37596768 PMC10524059

[21] Spitzer RL , Kroenke K, Williams JBW, Löwe B. A brief measure for assessing generalized anxiety disorder: the GAD-7. Arch Intern Med. 2006;166(10):1092-1097. doi:10.1001/archinte.166.10.1092 16717171

[22] Kroenke K , Spitzer RL, Williams JBW. The PHQ-9. J Gen Intern Med. 2001;16(9):606-613. doi:10.1046/j.1525-1497.2001.016009606.x 11556941 PMC1495268

[23] The Generalized Anxiety Disorder Screener (GAD-7) and the anxiety module of the Hospital and Depression Scale (HADS-A) as screening tools for generalized anxiety disorder among cancer patients—PubMed. https://pubmed.ncbi.nlm.nih.gov/29473255/. Accessed December 9, 2021. 10.1002/pon.4681 29473255

[24] Hartung TJ , Friedrich M, Johansen C, et al The Hospital Anxiety and Depression Scale (HADS) and the 9-item Patient Health Questionnaire (PHQ-9) as screening instruments for depression in patients with cancer. Cancer. 2017;123(21):4236-4243. doi:10.1002/cncr.30846 28654189

[25] Kroenke K , Strine TW, Spitzer RL, Williams JBW, Berry JT, Mokdad AH. The PHQ-8 as a measure of current depression in the general population. J Affect Disord. 2009;114(1-3):163-173. doi:10.1016/j.jad.2008.06.026 18752852

[26] Wu Y , Levis B, Riehm KE, et al Equivalency of the diagnostic accuracy of the PHQ-8 and PHQ-9: a systematic review and individual participant data meta-analysis. Psychol Med. 2020;50(8):1368-1380. doi:10.1017/S0033291719001314 31298180 PMC6954991

[27] Sun H , Yang Y, Zhang J, et al Fear of cancer recurrence, anxiety and depressive symptoms in adolescent and young adult cancer patients. Neuropsychiatr Dis Treat. 2019;15:857-865. doi:10.2147/NDT.S202432 31118635 PMC6498985

[28] Geue K , Brähler E, Faller H, et al Prevalence of mental disorders and psychosocial distress in German adolescent and young adult cancer patients (AYA). Psychooncology. 2018;27(7):1802-1809. doi:10.1002/pon.4730 29644783

[29] Desai MJ , Gold RS, Jones CK, et al Mental health outcomes in adolescent and young adult female cancer survivors of a sexual minority. J Adolesc Young Adult Oncol. 2021;10(2):148-155. doi:10.1089/jayao.2020.0082 32730111 PMC8064930

[30] National Center for Health Statistics. *NHIS—About the National Health Interview Survey*. https://www.cdc.gov/nchs/nhis/about_nhis.htm. Published September 16, 2020. Accessed November 30, 2021.

[31] Blewett LA , Rivera Drew JA, King ML, Williams KC. *Natalie Del Ponte, Pat Convey. IPUMS Health Surveys: National Health Interview Survey, Version 7.1 [dataset].* 10.18128/D070.V7.1. Accessed November 22, 2021.

[32] *NHIS—Methods*. https://www.cdc.gov/nchs/nhis/methods.htm. Published June 14, 2021. Accessed December 9, 2021.

[33] *IPUMS NHIS*. https://nhis.ipums.org/nhis/userNotes_variance.shtml. Accessed June 9, 2022.

[34] Karabon P. *Applying Propensity Score Methods to Complex Survey Data Using PROC PSMATCH.* https://support.sas.com/resources/papers/proceedings19/3634-2019.pdf. Accessed July 14, 2023.

[35] Marchak JG , Christen S, Mulder RL, et al; International Guideline Harmonization Group psychological late effects group. Recommendations for the surveillance of mental health problems in childhood, adolescent, and young adult cancer survivors: a report from the International Late Effects of Childhood Cancer Guideline Harmonization Group. Lancet Oncol. 2022;23(4):e184-e196. doi:10.1016/S1470-2045(21)00750-6 35358467 PMC9639707

[36] National Comprehensive Cancer Network. *NCCN Clinical Practice Guidelines in Oncology: Survivorship. 2022; Version 1*. 2022. https://www.nccn.org/professionals/physician_gls/pdf/survivorship.pdf. Accessed March 30, 2022.

[37] Andersen BL , Lacchetti C, Ashing K, et al Management of anxiety and depression in adult survivors of cancer: ASCO guideline update. J Clin Oncol Off J Am Soc Clin Oncol. 2023;41(18):3426-3453. doi:10.1200/JClinOncol.23.00293 37075262

[38] U.S. Preventive Services Task Force. Screening for depression in adults: U.S. Preventive Services Task Force recommendation statement. Ann Intern Med 2009;151(11):784-792. doi:10.7326/0003-4819-151-11-200912010-00006 19949144

[39] Barry MJ , Nicholson WK, Silverstein M, et al; US Preventive Services Task Force. Screening for anxiety disorders in adults: US Preventive Services Task Force recommendation statement. JAMA. 2023;329(24):2163-2170. doi:10.1001/jama.2023.9301 37338866

