# Supplementary material for: Mental health and care utilization in survivors of adolescent and young adult cancer
Source: JNCI Cancer Spectr. 2023 Nov 20;7(6):pkad098. doi: 10.1093/jncics/pkad098 (PMC10735415; doi:10.1093/jncics/pkad098)
Supplement: pkad098_Supplementary_Data [file pkad098_supplementary_data.pdf]

Supplementary Material

Supplementary Table 1: Cancer Type Frequencies
Supplementary Table 2: Regression Model Output, with Crude and Adjusted Incidence Rate or Odds Ratios, according to Model Utilized Excluding AYA survivors with Time Since Diagnosis <1 Year
Supplementary Figure 1: Distribution of GAD7 Score
Supplementary Figure 2: Distribution of PHQ8 Score
Supplementary Figure 3: Graphical Output from Propensity Score Weighting
Supplementary Figure 4: Distribution of Time Since Diagnosis
Supplementary Figure 5: Distribution of Age at Diagnosis
Supplementary Figure 6: Structural Equation Models of Adolescent and Young Adult Cancer Survivorship, Symptom Severity, and Care Utilization Excluding AYA survivors with Time Since Diagnosis <1 Year

Supplementary Table 1: Frequency of Cancer Types Among Adolescent and Young Adult Cancer Survivors

Cancer Type	Unweighted Count	Weighted %
Hematologic	39.00	8.45
Bone	8.00	1.43
Brain	12.00	2.04
Breast	47.00	7.76
GYN	179.00	26.47
Colorectal	9.00	1.53
Other GI	3.00	0.46
Head & Neck	3.00	0.67
Lung	4.00	0.52
Melanoma	82.00	11.72
Non-Melanoma Skin	124.00	17.63
GU	20.00	2.23
Thyroid	38.00	6.28
Other	71.00	12.81

Supplementary Table 2: Regression Model Output, with Crude and Adjusted Incidence Rate or Odds Ratios, according to Model Utilized Excluding AYA survivors with Time Since Diagnosis <1 Year.

Model	Independent Variable	Dependent Variable	n^	Crude	Adjusted[+]
Negative Binomial Models				*Incidence Rate Ratio (95% CI)*	
1	AYA Survivorship	PHQ8 Total Score	30,432	1.37 (1.09-1.73)**	1.44 (1.10-1.89)**
2	AYA Survivorship	GAD7 Total Score	30,432	1.39 (1.10-1.75)**	1.74 (1.32-2.28)***
3	Time Since Diagnosis	PHQ8 Total Score	639	1.00 (0.99-1.01)	0.99 (0.98-0.99)**
4	Time Since Diagnosis	GAD7 Total Score	639	0.98 (0.97-0.99)**	0.98 (0.97-0.99)***
Logistic Regression Models				*Odds Ratio (95% CI)*	
5	AYA Survivorship	Therapy Use	30,432	1.28 (0.91-1.79)	2.00 (1.19-3.36)**
6	AYA Survivorship	Medication Use	30,432	1.40 (1.04-1.88)*	1.95 (1.16-3.26)*
7	Time Since Diagnosis	Therapy Use	639	0.99 (0.97-1.01)	0.95 (0.93-0.98)**
8	Time Since Diagnosis	Medication Use	639	0.99 (0.97-1.01)	0.95 (0.93-0.98)**

Supplementary Figure 1: Distribution of Generalized Anxiety Disorder 7 (GAD-7) Total Scores

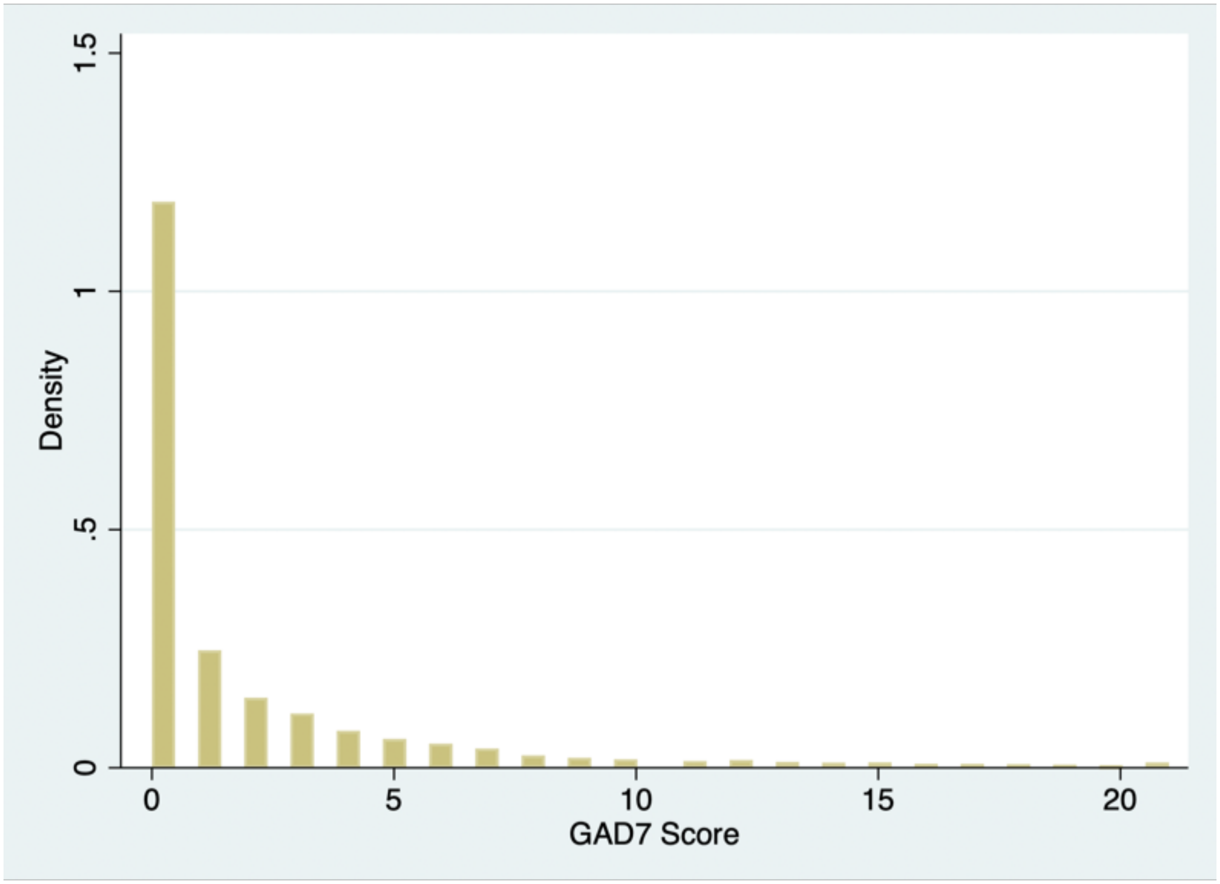

Supplementary Figure 2: Distribution of Patient Health Questionnaire 8 (PHQ8) Total Scores

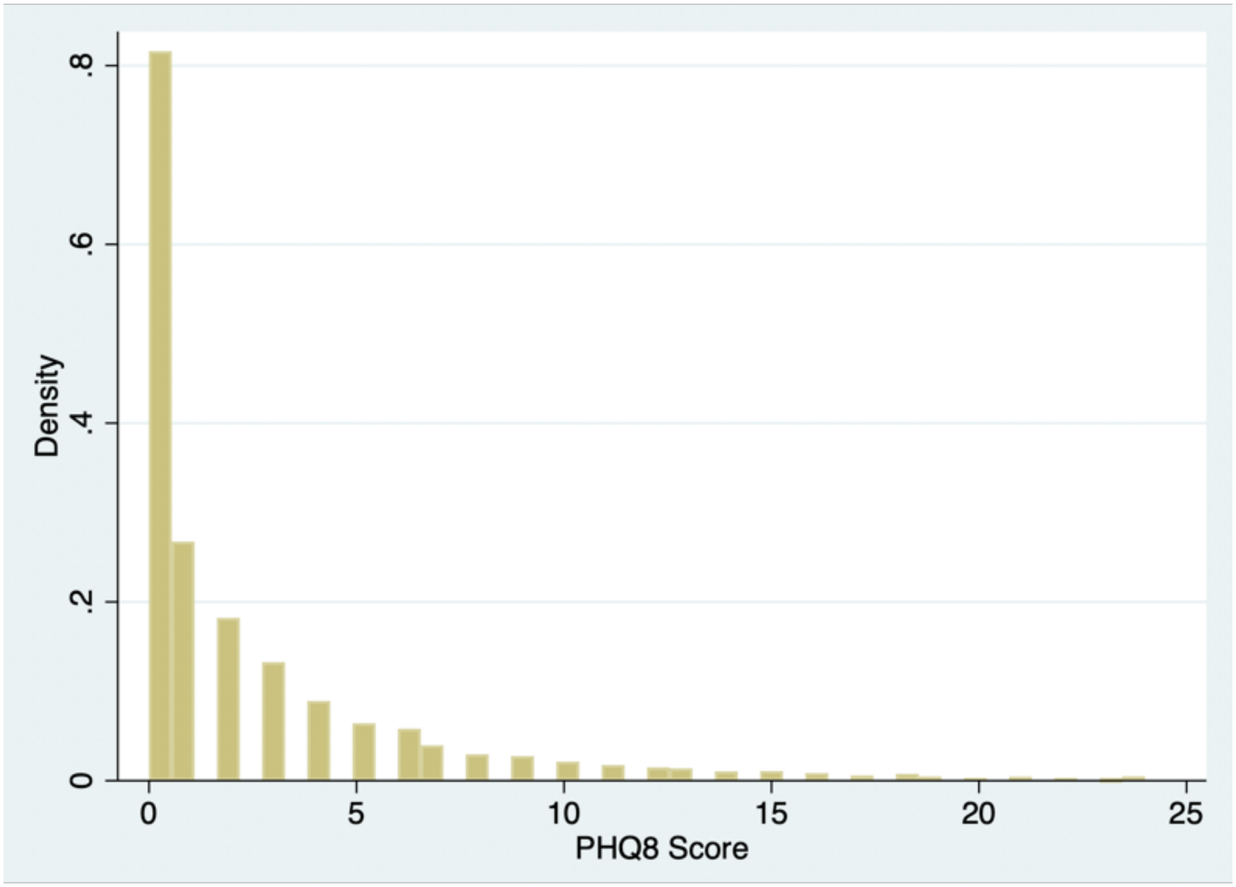

Supplementary Figure 3: Graphical Output from Propensity Score Weighting

a. Standardized Mean Differences

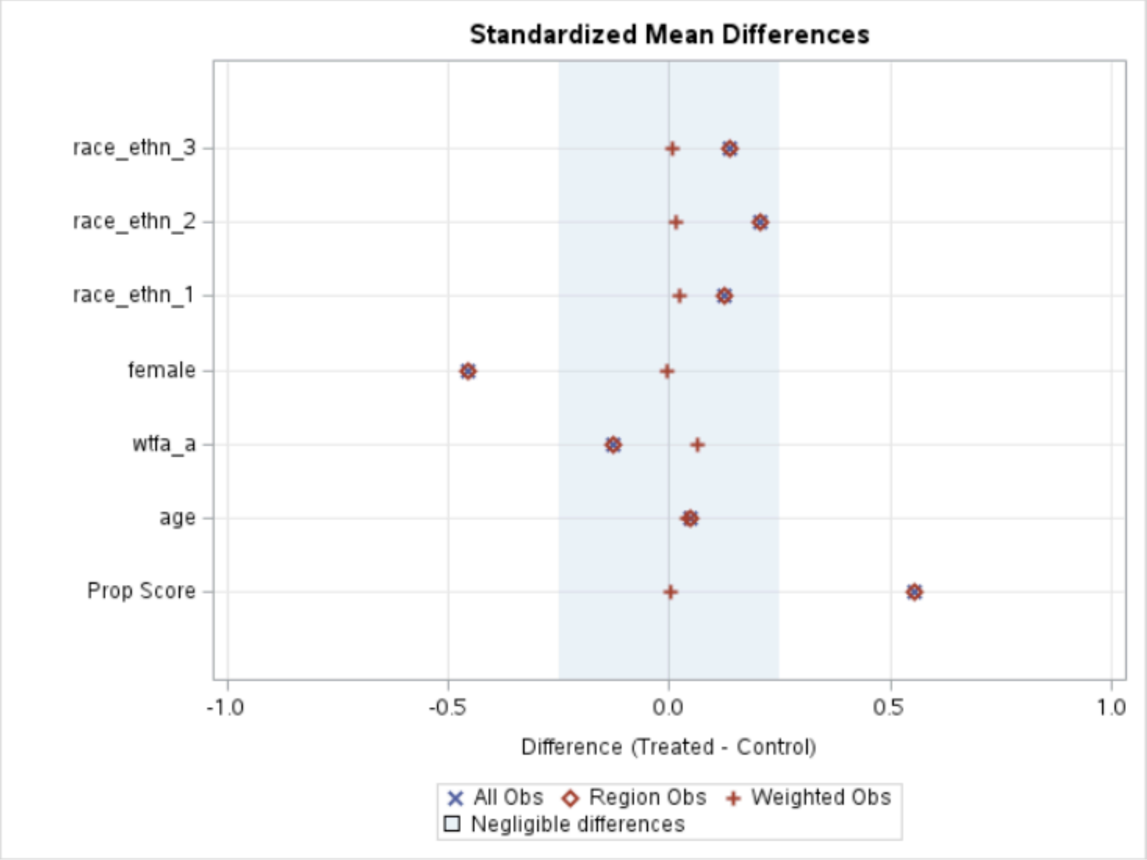

Graphical representation of standardized mean differences between exposure group (AYA cancer survivors) and comparison group (general population without history of AYA cancer) before and after applying propensity weights. X indicates standardized mean difference for all observations prior to application of inverse propensity score weight. ◇ indicates observations in specified region, which for our analysis was all observations. + indicates standardized mean differences once inverse propensity score weights were applied. Variable balance is best when post-weighting standardized mean differences are close to 0, but a widely accepted benchmark for successful propensity score weighting is mean differences between -0.5 and 0.5. Y axis variables are right-sided variables (age, female = sex, race_ethn = race/ethnicity, wtfa_a = survey weights) and outcome (propensity score) in the propensity score model. Race/Ethnicity variables are dummy coded for 3 non-referent levels of the variable (race_ethn_1: Hispanic, race_ethn_2: Non-Hispanic Black, race_ethn_3: Other and Multiple Race). Referent level was Non-Hispanic White.

b. Distribution of Propensity Scores

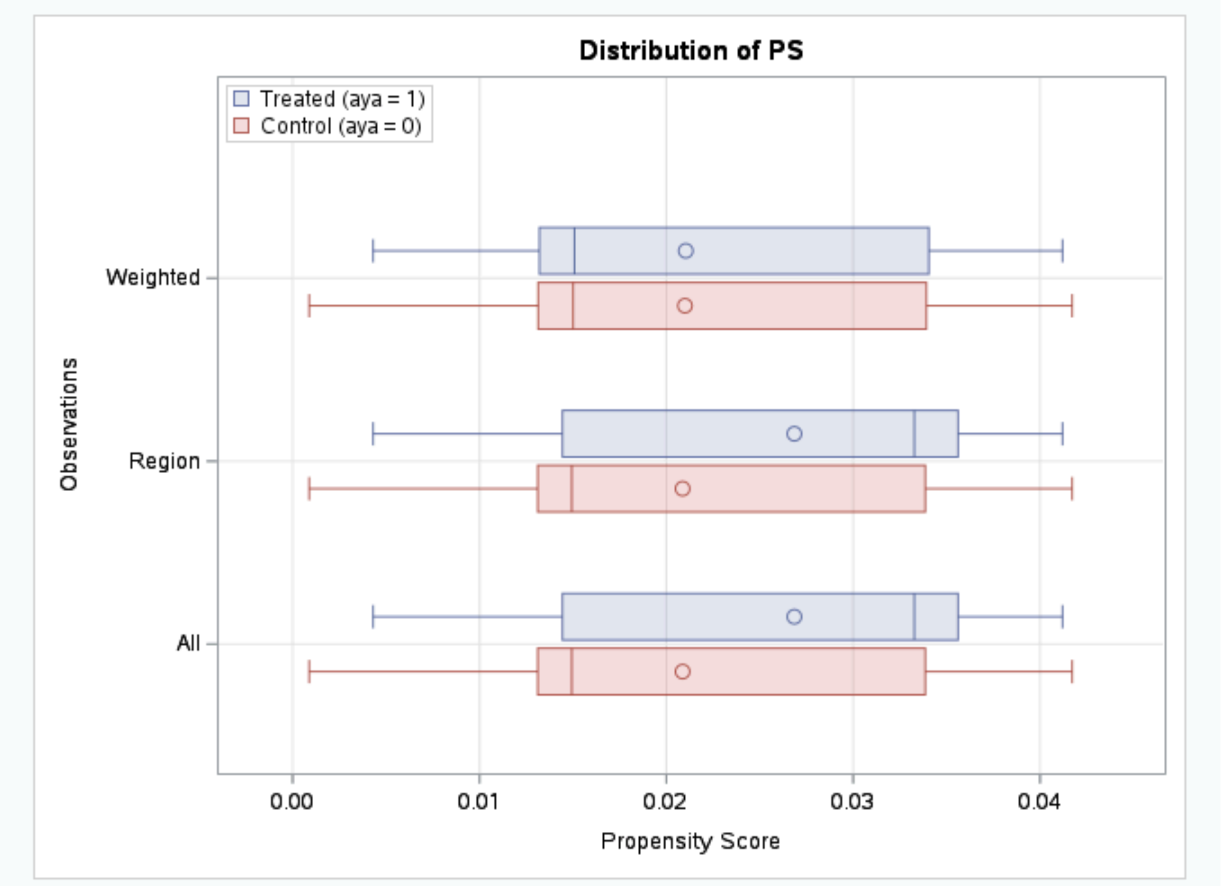

All: all observations pre-weighting. Region: pre-specified region, which for our analysis was all observations, pre-weighting. Weighted: post-weighting. PS: Propensity Score.

c. Distribution of Age Variable

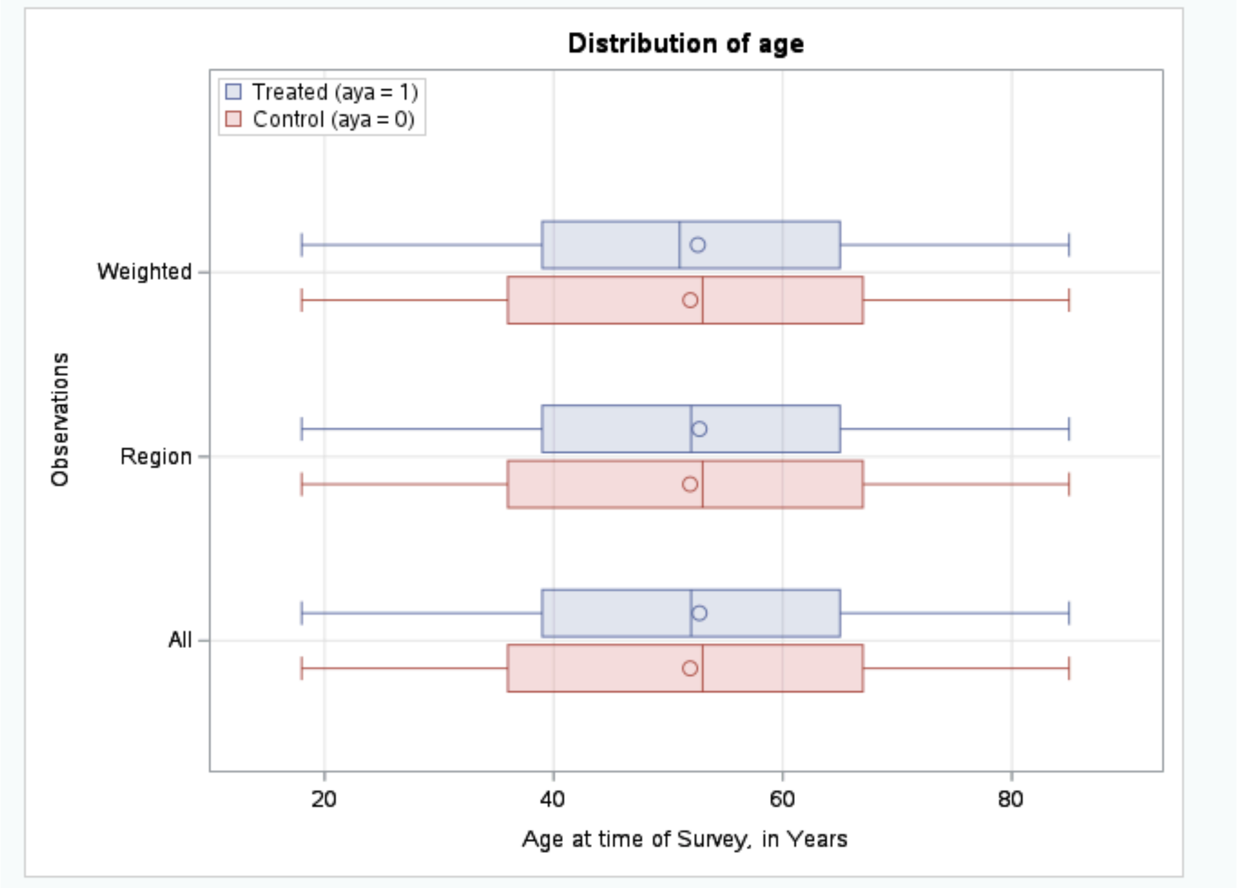

All: all observations pre-weighting. Region: pre-specified region, which for our analysis was all observations, pre-weighting. Weighted: post-weighting.

d. Distribution of Sex Variable

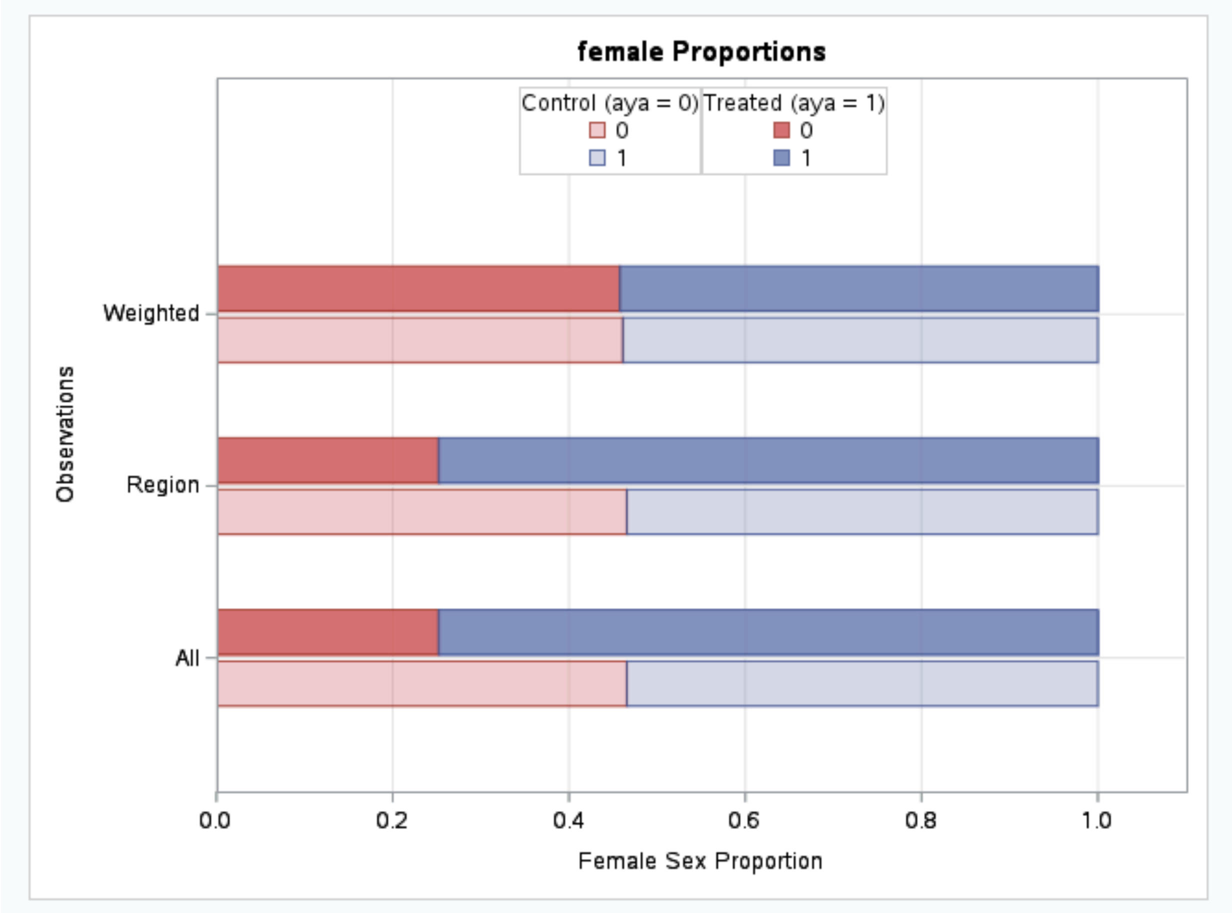

All: all observations pre-weighting. Region: pre-specified region, which for our analysis was all observations, pre-weighting. Weighted: post-weighting. Female: variable name for sex where female=1 for sex=female, female=0 for sex=male.

e. **Distribution of Race/Ethnicity Variable Level 1**

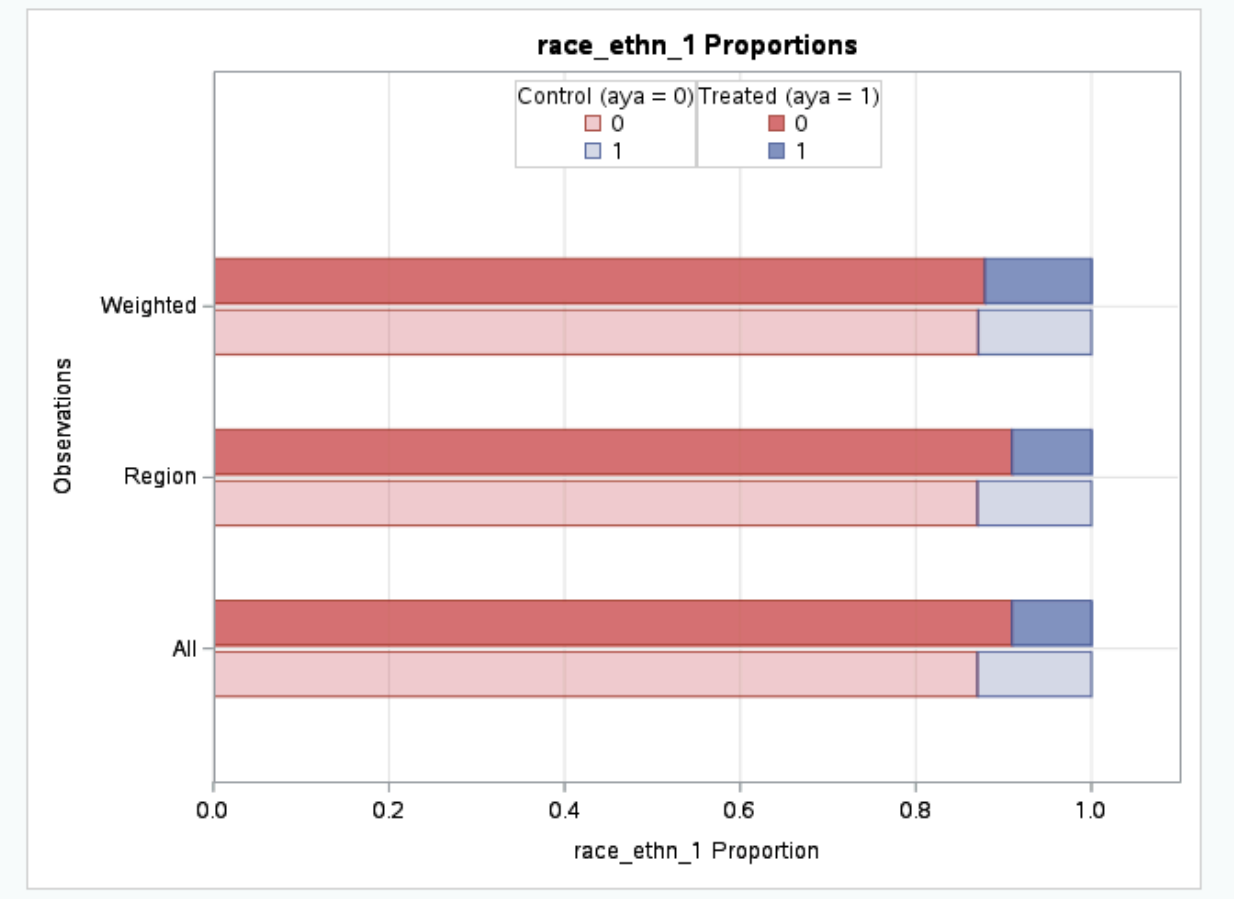

All: all observations pre-weighting. Region: pre-specified region, which for our analysis was all observations, pre-weighting. Weighted: post-weighting. race_ethn_1: dummy variable for Hispanic where race_ethn_1=1 corresponds to Hispanic and race_ethn_1=0 to not Hispanic.

f. Distribution of Race/Ethnicity Variable 2

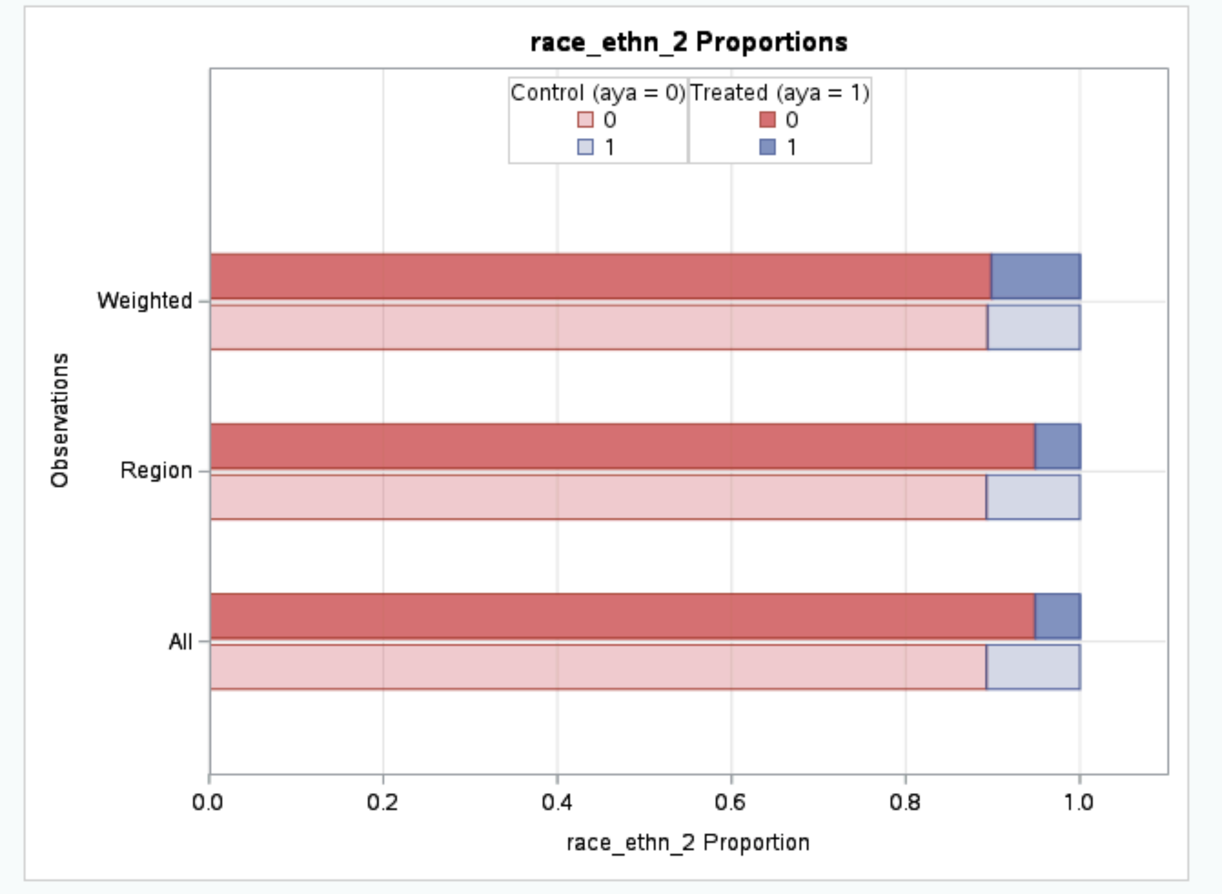

All: all observations pre-weighting. Region: pre-specified region, which for our analysis was all observations, pre-weighting. Weighted: post-weighting. race_ethn_2: dummy variable for Non-Hispanic Black where race_ethn_2=1 corresponds to Non-Hispanic Black and race_ethn_1=0 to not Non-Hispanic Black.

g. Distribution of Race/Ethnicity Variable 3

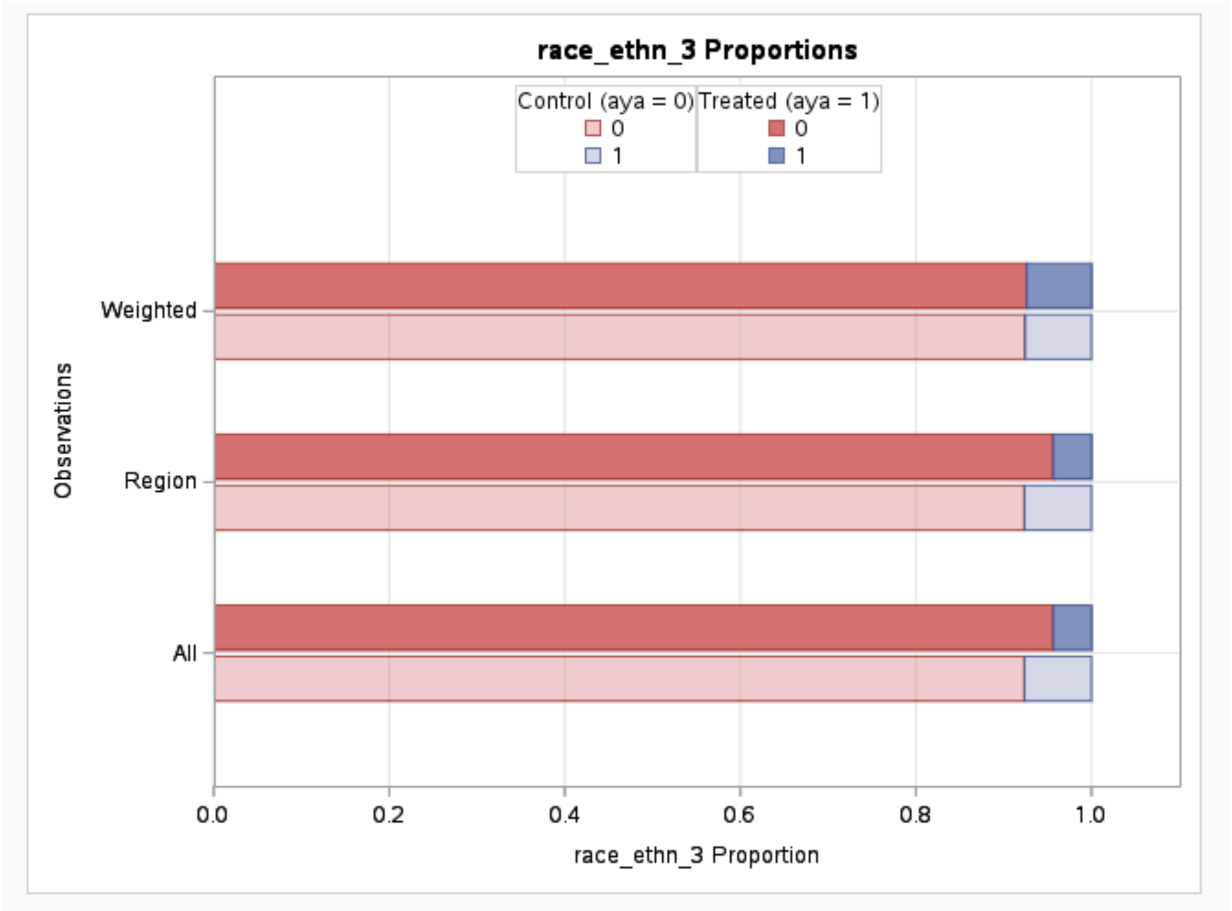

All: all observations pre-weighting. Region: pre-specified region, which for our analysis was all observations, pre-weighting. Weighted: post-weighting. race_ethn_3: dummy variable for Other and Multiple Race where race_ethn_3=1 corresponds to Other and Multiple Race and race_ethn_3=0 to not Other and Multiple Race.

Supplementary Figure 4: Distribution of Time Since Diagnosis Among Adolescent and Young Adult Cancer Survivors

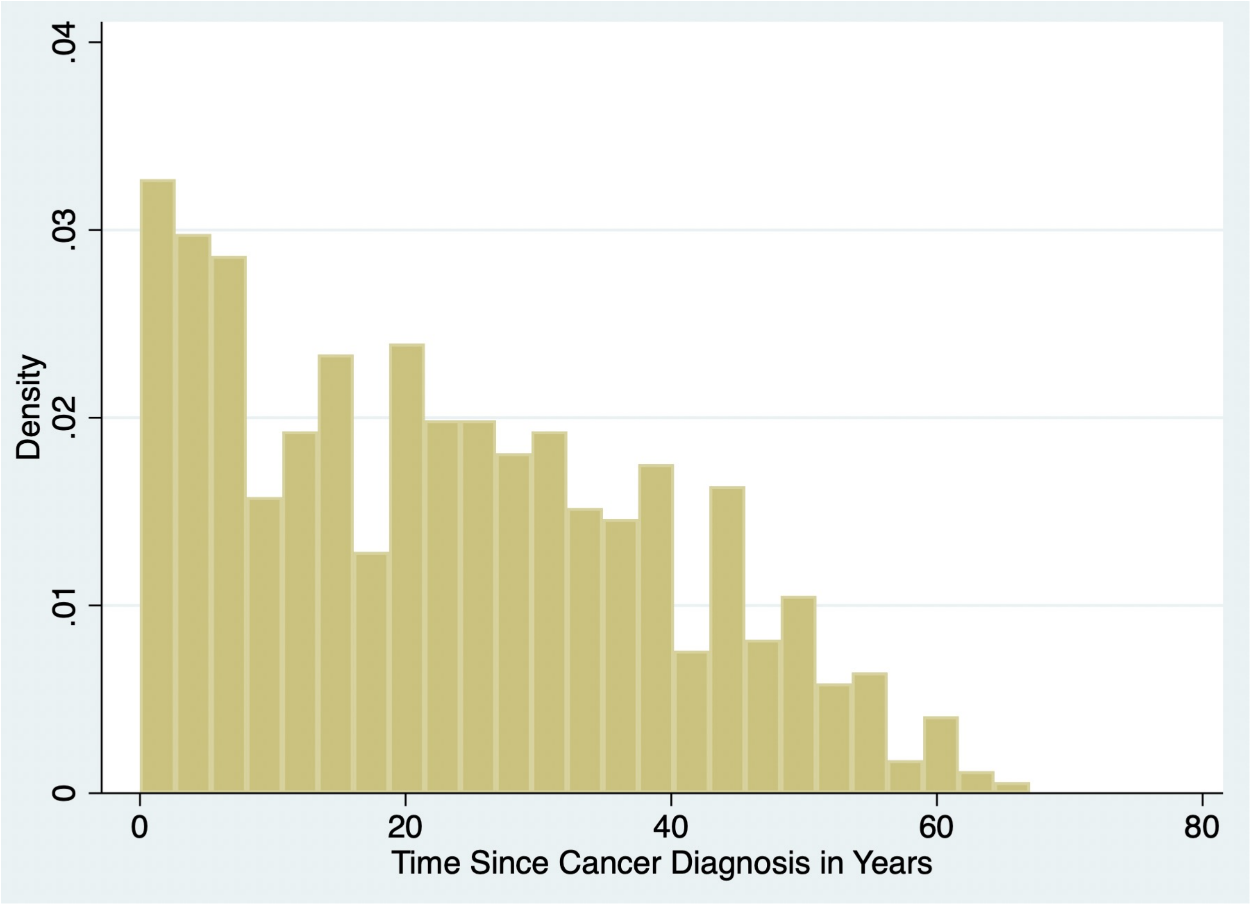

Supplementary Figure 5: Distribution of Age at First Cancer Diagnosis Among Adolescent and Young Adult Cancer Survivors

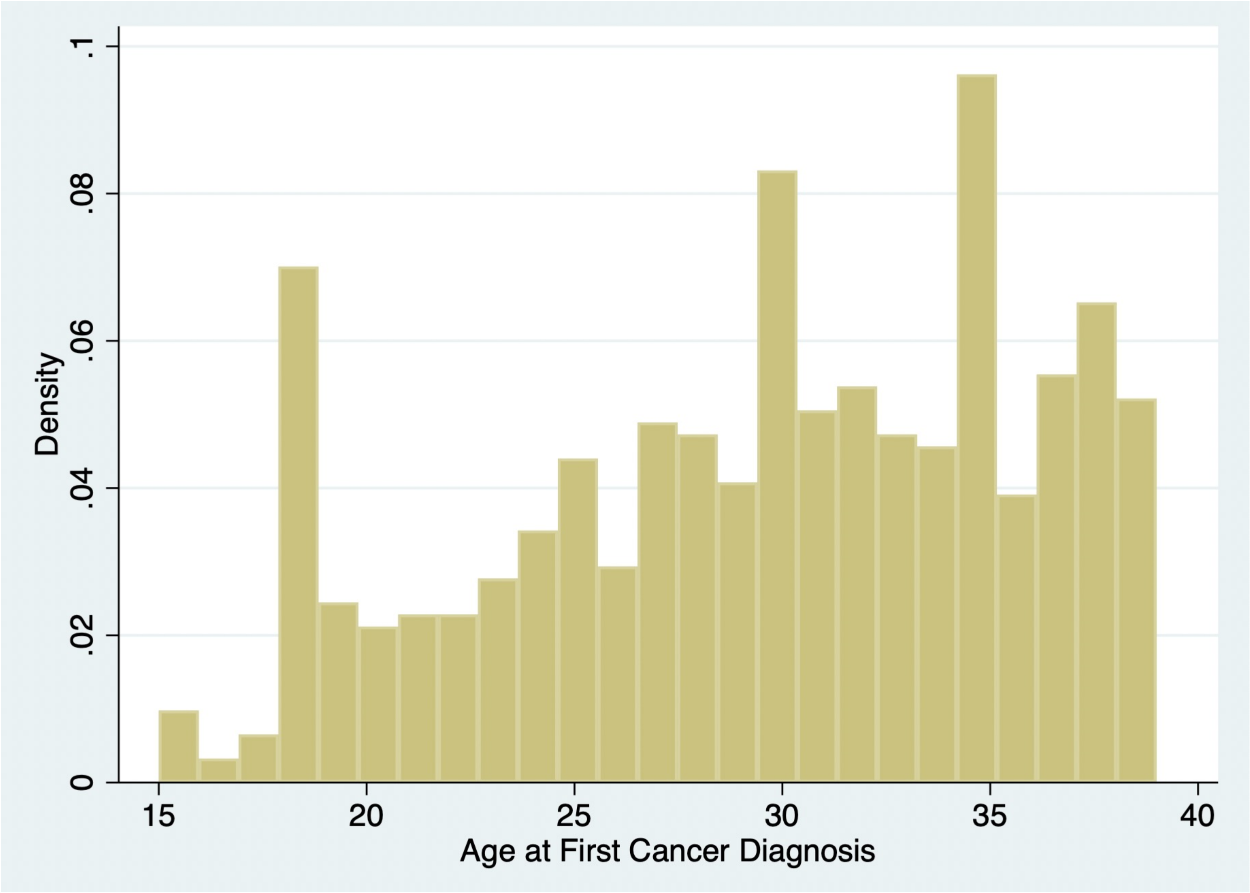

Supplementary Figure 6: Structural Equation Models of Adolescent and Young Adult Cancer Survivorship, Symptom Severity, and Care Utilization Excluding AYA survivors with Time Since Diagnosis <1 Year

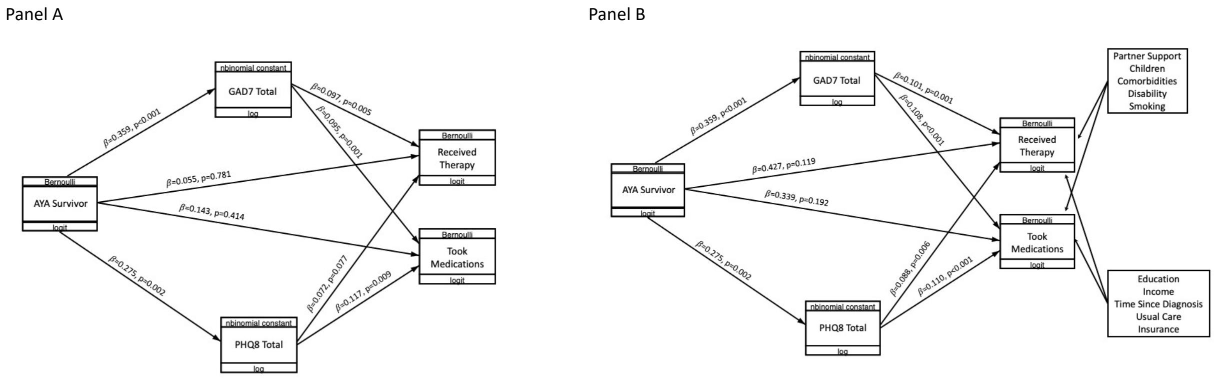

Panel A. Unadjusted Structural Equation Model (SEM) with mediated (indirect) and residual (direct) pathways from AYA Survivor (AYA cancer survivors, referent no history of AYA cancer) to healthcare utilization variables – Received Therapy (received psychotherapy in the last 12 months, referent no psychotherapy) and Took Medications (received mental health medications in the last 12 months, referent no medication use). Individual variables top-labelled with generalized linear model family (ex: Bernoulli) and bottom-labelled with link function (ex: logit). β values represent GLM regression coefficients with associated p-values.
Panel B. SEM adjusted for selected covariates.
AYA: Adolescent and Young Adult. GAD7: Generalized Anxiety Disorder 7. PHQ8: Patient Health Questionnaire 8.
